# “I attend at Vanguard and I attend here as well”: barriers to accessing healthcare services among older South Africans with HIV and non-communicable diseases

**DOI:** 10.1186/s12939-018-0863-4

**Published:** 2018-09-18

**Authors:** Lucia Knight, Enid Schatz, Ferdinand C. Mukumbang

**Affiliations:** 1 School of Public Health, University of the Western Cape, P Bag X17, Bellville, 7535 South Africa; 2 Department of Health Sciences and Department of Women’s & Gender Studies, University of Missouri, Columbia, Missouri USA

**Keywords:** South Africa, Chronic disease care, HIV, Older persons, Health services, Health policy, Syndemic

## Abstract

**Background:**

HIV and non-communicable disease (NCD) are syndemic within sub-Saharan Africa especially among older persons. The two epidemics interact with one another within a context of poverty, inequality and inequitable access to healthcare resulting in an increase in those aged 50 and older living with HIV and experiencing an NCD co-morbidity. We explore the challenges of navigating healthcare for older persons living with HIV and NCD co-morbidity.

**Methods:**

In-depth semi-structured interviews were conducted with a small sample of older persons living with HIV (OPLWH). The perspectives of key informants were also sought to triangulate the evidence of OPLWH. The research took place in two communities on the outskirts of Cape Town, South Africa. All interviews were conducted by a trained interviewer and transcribed and translated for analysis. Thematic content analysis guided data analysis.

**Results:**

OPLWH experienced an HIV-NCD syndemic. Our respondents sought care and accessed treatment for both HIV *and* other chronic (and acute) conditions, though these services were provided at different health facilities or by different health providers. Through the syndemic theory, it is possible to observe that OPLWH and NCDs face a number of physical and structural barriers to accessing the healthcare system. These barriers are compounded by separate appointments and spaces for each condition. These difficulties can exacerbate the impact of their ill-health and perpetuate structural vulnerabilities. Despite policy changes towards integrated care, this is not the experience of OPLWH in these communities.

**Conclusions:**

The population living with HIV is aging increasing the likelihood that those living with HIV will also be living with other chronic conditions including NCDs. Thus, it is essential that health policy address this basic need to integrate HIV and NCD care.

## Background

The aging of the South African population is occurring in the context of a double burden of disease –high HIV prevalence and an emerging epidemic of non-communicable diseases (NCD) [1–7]. Importantly, the co-incidence of these two epidemics (HIV and NCDs) suggests a high proportion of co-morbidity and that these two conditions are syndemic [8, 9]. The syndemic model proposes the aggregation of two or more concurrent epidemics with biologic interaction, which exacerbates the prognosis and burden of both diseases. According to Singer et al. ([10]: 942) “The criteria of a syndemic are: (1) two (or more) diseases or health conditions cluster within a specific population; (2) contextual and social factors create the conditions in which two (or more) diseases or health conditions cluster; and (3) the clustering of diseases results in adverse disease interaction, either biological or social or behavioural, increasing the health burden of affected populations.”

Biological aging resulting in immunosenescence and frailty may heighten vulnerability to HIV, other infectious diseases and NCDs [11–15]. Older persons (over 50 years) living with HIV are potentially also more at risk of other chronic conditions, either due to being-HIV positive or as a result of taking antiretroviral treatment [12, 16–18]. Beyond the biological and individual factors (age, treatment) that interact to result in this syndemic, older persons within Southern Africa are also potentially vulnerable because of social and economic conditions such as poverty, poor living conditions and other structural factors [10, 19]. The need for research about and consideration of aging and HIV in low and middle income contexts is increasingly recognised [20], as is the relevance of exploring the linkages between HIV and NCDs [21, 22].

Despite the high prevalence of co-morbidities in older persons, there is limited evidence examining the impact of the health system on the syndemic, including healthcare access and targeted treatment modalities for OPLWH who have additional chronic conditions. Evidence from rural settings in Uganda and South Africa suggests that OPLWH with co-morbidities access health care more regularly, and with potentially better health outcomes, than those with single chronic conditions [23–25]. Sometimes called an antiretroviral therapy (ART)-advantage, the attachment to health services due to HIV may explain why OPLWH are more likely to know if they have additional chronic conditions and to have it treated, compared to those without HIV [25, 26]. Chronic communicable disease, including HIV, has been found to be an important positive predictor of older persons’ healthcare usage and having HIV and being on ART was a positive predictor of being cared for another NCD in two rural South Africa studies [27, 28]. OPLWH in rural Uganda had visited a clinic more recently that those who were HIV-negative; these visits were for both HIV and NCD care [29].

These findings support an increasing recognition of the need for greater integration in the health services of HIV and NCDs [30–32], as OPLWH are likely to be seeking care for both. Within South Africa, historically, the services for each are delivered vertically, and therefore, likely segmented or bifurcated in a range of ways [30, 33, 34]. The HIV and NCD syndemic poses complex challenges for access to care in an already taxed healthcare system and there is potential for the syndemic to be exacerbated by problems with access to care and treatment [9, 10]. To respond to the health needs of older South Africans, it is important to explore the experiences of OPLWH in accessing healthcare and treatment for co-morbidities. Therefore, in this paper, we use qualitative interviews in two urban communities in Cape Town to explore in-depth how the healthcare seeking experiences of OPLWH may contribute to exacerbating this syndemic.

## Methods

We used an exploratory qualitative study design to examine the experiences of access to care for OPLWH with NCDs in two urban communities, Langa and Khayelitsha, in the outskirts of Cape Town. The communities of Langa and Khayelitsha are predominantly black townships. Langa is located 10 miles outside of Cape Town while Khayelitsha is a little further beyond the Cape Town city limits. Both communities are a mix of formal and informal settlements, although Khayelitsha is larger and more dominated by informal housing especially near the clinic under study. These communities are poor and have a majority of unemployed inhabitants with many social problems [35]; however, both communities have relatively good access to health services [36].

### Study setting: Healthcare services

Langa has an established clinic situated at the centre of the community allowing for relatively easy access for residents. This government-run primary health care facility provides maternity services to pregnant women and HIV, AIDS and TB-related treatment, care and support services. The Vanguard Community Health Centre (CHC), a second health facility used by residents of Langa, is situated in Bonteheuwel, another community located across the main road. The CHC is also a government-run facility providing similar services to the Langa Clinic. It also provides adult curative acute and chronic care services. The CHC is about 2.5 km away from the Langa Clinic and it takes about 30 min to walk between the two facilities.

Khayelitsha is serviced by different clinics and community health centres but this study is focused on OPLWH seeking care at the Khayelitsha Site B Community Health Clinic. Site B provides the same services as Vanguard CHC, which include HIV counselling and testing (HCT), an ART clinic and adult curative and chronic care services. Like Vanguard, at Site B different members of staff provide HCT, ART and curative and chronic care services in different sections (units). Services at both facilities are subsidized and there is limited or no direct cost for the services.

### Study sample

Our sampling approach was purposive with the goal of variation among our study participants. This required employing creative sampling strategies; (1) we consulted a survey list from a local NCD research project, which included a self-report question about HIV status, (2) we connected to respondents through contacts at the HIV clinics and (3) we found respondents through convenience sampling at clinics. The range of sampling techniques provided us with an opportunity to identify respondents who fit the selection criteria - being an older person (age 50 or greater) living with HIV. We attempted to include a similar number of men and women in the sample as well as respondents representing a range of ages over 50. Table 1 displays the characteristics of the individuals who participated in the study.

**Table 1:** Representation of study participants from the two sites by sex and age

Characteristics	Khayelitsha	Langa	Total
Sex			
Male	9	4	13
Female	5	5	10
Age			
50–54	0	4	4
55–59	4	4	8
60–64	7	1	8
65 +	3	0	3

To triangulate data and ensure rigor, we conducted key informant interviews (KII) with ten service providers to gain a broader perspective on the issues facing OPLWH. The sample included: five staff working at the primary health facilities where the respondents in the study sought HIV and NCD care, one home based care-giver, two social workers, one senior (old age) club employee, and one employee at South Africa Social Security Agency (SASSA).

### Data collection

Data were collected from the OPLWH and key informants using semi-structured in-depth qualitative interviews. Interview guides were developed based on the evidence from existing research and informed by the research questions. The qualitative nature of the research means that the tools were guides only and that the topics covered in all interviews were adapted to the individual. The interviews were conducted in isiXhosa or where possible and requested in English by a trained and experienced qualitative interviewer who herself is an older person from a community similar to those in the study. The interviews were focused on the older persons’ experiences of living with HIV. The interviews with both OPLWH and key informants included particular questions about older persons’ experiences of their access to healthcare for their HIV and any co-morbidities and if required probes about the various factors that they felt either facilitated or were barriers to this. The interviews for OPLWH mostly took place in participants’ homes. The KII and a few of the OPLWH interviews took place in a quite space within the facility or the relevant place of work where people felt comfortable and privacy could be ensured.

All interviews were audio-recorded and fully transcribed, where the interviews were conducted in isiXhosa the interviews were simultaneously translated and transcribed in English from isiXhosa by the interviewer.

### Data analysis

Adopting a thematic content analysis approach [37], we inductively coded the data. We (1) read the transcripts several times for familiarisation, (2) coded the data by identifying key points and recurrent emergent issues regarding access to care by OPLWH, and (3) identified and organised the codes into themes. The codes and themes were based on participants’ experiences of having a comorbidity and access to care. For the KII, codes and themes included the perspectives of the KII about access to care for OPLWH with other chronic conditions. The data analysis was managed using NVivo version 10.0. To ensure rigor the transcripts were read and coded by ES and LK and the analysis was discussed as a team.

## Results

All 23 of the participants were living with HIV and had initiated ART – some just a couple months prior to being interviewed, while others had been on ART for many years. The results highlight the participants’ experiences of accessing treatment and healthcare in the context of co- or multi-morbidity. All of our respondents had at least one co-morbidity. A number of the participants had been or were being treated for TB. The majority of those who reported second or additional health conditions, other than HIV, experienced chronic conditions, including high blood pressure, arthritis, and diabetes. A few participants reported other conditions that required ongoing engagement with the healthcare system such as mental health problems, stroke and physical disabilities.

In this section, the experience of accessing treatment is explored using the qualitative data. Proximity, travel costs and time to access care emerged as a sub-theme.

### Accessing treatment for multiple health conditions

The South African Public Health service conceived of and delivers HIV treatment and care as a vertical program [30]. Treatment for common HIV co-morbidities, particularly TB, is integrated with HIV care [38]. Therefore, respondents in this study accessed and received TB and HIV treatment together at an HIV clinic, in Langa or Khayelitsha. Treatment or care for other chronic conditions, however, which often entailed regular appointments, required accessing different health services or providers. All respondents from Langa accessed their ART at the Langa HIV clinic; the services for the management of and access to treatment for other chronic conditions were provided at the Vanguard Community Health Centre, about 2.5 km (minimum 30 min’ walk) from the Langa Clinic. An older man from Langa described where he accesses treatment:

*It’s the one that is here at Langa* ***and*** *the one that is at Vanguard. Here at Langa, I get the one [treatment] to prevent TB and the one for AIDS… and then I get the one [treatment] for my brain [mental illness] up there at Vanguard. (58 year-old man, Langa)*

The Langa HIV clinic staff supported this claim by describing the facility’s referral process,

*You know we also have quite a few clients that test positive [for HIV] now, over the age of sixty, but we only treat HIV that is chronic, [but not other chronic conditions]. We do diagnose here, like hypertension, diabetes and then we refer to Vanguard, the day hospital. (HIV clinic nurse, Langa)*

‘Chronic’ HIV refers to ongoing treatment and care received after an HIV diagnosis; other diagnoses include referrals to another facility. In Khayelitsha, although the facilities for HIV and chronic adult illness are physically located in the same complex, the services are separate and clients receive their care and treatment for HIV and chronic conditions in two different places. One older man described his situation as such,

*…I get [all my treatment] from this clinic, just like now, I have come to fetch these pills for HIV today, tomorrow is my due date there in front [of the clinic at the Out Patients Department (OPD)] for High Blood [Pressure treatment]. Then I get this one [HIV medication] here [at the HIV clinic]. No, it doesn’t happen, I haven’t had it yet [that the dates for the appointments coincide]. My appointments are separate. (67 year-old man, Khayelitsha)*

The experience of clients at Khayelitsha, like those in Langa, suggest bifurcated care for HIV and co-morbidities. Not only were services provided separately, but providers also did not link or coordinate the appointments for HIV and other chronic conditions.

In Khayelitsha, linked appointments would occur together or at least consecutively in 1 day. In Langa, coordination of HIV and NCD services would necessitate appointments *not* be scheduled on the same day due to the distance between the facilities. The Langa respondents provided examples of having had clashing appointments for their HIV and chronic care,

*I do it but it challenges me because on the other side [Vanguard CHC] I arrive late because I start on this side [at Langa Clinic]…sometimes their dates clash, the one here [at Langa clinic] and the one for Vanguard…then I start here and arrive late over there. There I then tell them the reason for being late: that I started there… then they help me but at other times you miss out because you are told the doctors have left…perhaps you had to meet with the doctor there, and I don’t find the doctor there now. Yes, you are given another appointment. (55 year-old woman, Langa)*

Another respondent explained that missing her HIV appointment due to clashing or forgotten appointments was not as problematic as missing her NCD appointment. If she missed the latter she was more likely to run out of her treatments for high blood pressure and diabetes. She worried less that she would run out of ART if she missed her HIV appointment.

*…yet for those over there I don’t forget and here I do sometimes forget my date, the only thing is that they [HIV clinic] give a lot of pills [ART], they do that and it helps that they gave out a lot…. [I] go there [to Vanguard] having missed your date, they turn you back [away] and give you another date into the future…They punish you here [at Vanguard]…they turn you back and give you a date next week because you missed your date. (62 year-old woman from Langa)*

The HIV clinic nurse from Khayelitsha indicated that clients could ask for their appointments to be on the same day. She explained,

*It depends, if a person wants us to bring the dates together, we do that, we don’t have a problem because the person will say, “Sister [nurse], I am working, so I cannot manage to be here on Monday and be here again on Friday for my chronic medication.” So the best is to have one date, we are very accommodating here, we always ask them to first go to the day hospital [OPD] for your chronic medication, when you are done just come and collect your ARTs this side; that happens on the same day, we don’t have a problem. (HIV clinic nurse, Khayelitsha)*

The fact that patients must be the ones to request this arrangement is problematic, however, as many may not realize it is possible or feel empowered to ask.

For some respondents, getting care from two separate places led to confusion about where to seek what care modality. This was particularly the case among our respondents from Khayelitsha who had to navigate care from two sections in one facility. As one older man complained,

*Now I’ll have to find out whether I’ll be getting all my treatment here because I don’t know whether I should go to the other side or will I get it here. As far as I’m concerned this side treats this thing only and I have noticed that I’ve been transferred and I didn’t have the sense that here it could be given to me or should I go there when I go from here. (61 year-old man, Khayelitsha)*

Others echoed this confusion. Negotiating care for two conditions led to frustration for one older woman, who said,

*I got fed up, I was upset by another young man, he told me where to sit but didn’t tell me after that, that wasn’t the place to be. There was no movement from that side and time was running out, then I left and when I got to the other side, they asked me where I was all that time and I told them that I have been here for some time and that the young man at the card room told me to sit there. I told them I was upset about that then they attended to me. (70 year-old woman, Khayelitsha)*

The confusion and difficulty negotiating multiple appointments for different conditions were exacerbated when the older persons forgot their appointments.

*The thing that encourages me [to take and collect] my treatment is that if I miss that date, you struggle a lot the next day and there are certain questions that are asked as to why didn’t you fetch it yesterday. Then you realize that each day that you have to come to the clinic, you must come quickly and fetch those medicines. (67 year-old man, Khayelitsha)*

Access to care for ART and other treatment was challenging because these services were either physically or administratively separate, which meant needing to remember *and* get to multiple appointments.

### Proximity, travel costs and time

The problems with receiving treatment and care services in two places are compounded when one considers other barriers to accessing care. Our analysis revealed that distance to facilities affected the means by which older persons travelled, by foot or using public transportation, and thus the cost and/or time involved.

For the respondents from Langa, the barriers to accessing care at Vanguard for co-morbidities were greater than accessing HIV and TB care at the Langa Clinic. One initial barrier was the distance respondents needed to travel from their homes to the facility. The longer distance to Vanguard required the clients negotiating assistance or transportation. The facility was far (on average about 2.5 km), such that it required a minimum of a 30 min walk. This presented a particular challenge for those with physical difficulties and/or a disability. One older woman described her situation as such,

*…the [Vanguard CHC] is far, I struggle [to get there], you have to hire [a taxi] when you go to the clinic because not all of us have cars, we have to hire [taxis] when we go to the clinic. We don’t even have the money to pay for a car [taxi] at that time; pension comes on the first [of the month] and then perhaps [the appointment] at this time [is] on the 18th/17th…the [pension] money is now finished, you have to go to the clinic and your date is here, there’s no money to pay. Now you have to ask for a car or you have to think… those are some of the things that cause stress because you think “God how am I going to go to the clinic?” (50 year-old woman from Langa)*

While public transport was available, making access to the facility relatively simple, affording this transport presented an impediment. Despite many of our respondents having access to the South African old age and disability social grants, which provide the equivalent of about USD120 per month, the cost of the shared minibus taxis was still an undue burden, particularly late in the month after much of the grant had been spent on other necessities. Further, overcrowding, long queues and time-consuming waiting times, particularly during peak travel times, made traveling by minibus taxis undesirable for older persons. An older woman explains her decision making process when considering how to travel to the facility for treatment.

*[I go] slowly on foot early in the morning [to Vanguard]…It is far but I go on foot until I get there there’s no problem but I get tired especially on the other side [at Vanguard]… You must slowly go on foot; with the taxis, you have to wait in a queue until it gets full…and then you realize that even that money…if you board a Delft [far off location] taxi because it goes past there you have to pay that amount paid to go to Delft but you are going around here… and yet even here at home, you need money for food, then you think no… (62 year-old woman, Langa)*

While many of our respondents in Khayelitsha lived close to the health facility, which reduced the distance and the cost of travel (key barriers), they still related these as barriers to accessing care. Being old made commuting tiring, which led them to either not make visits, or use taxis.

*Yes, they [both treatments] are obtainable there. I sometimes get High Blood, I become dizzy, and my head goes around. No, it’s me [who chooses not to go to the clinic], I’m just lazy to go there, and there is no reason…I’m lazy to go, it’s far. (59 year-old woman, Khayelitsha)*

She went on to say that when she felt ill or hungry, accessing the clinic was difficult, and complicated taking treatment.

*I usually board a taxi. Sometimes I go on foot… [but] sometimes I’m lazy to go [to the clinic] not having anything [to eat] here at home, having no food here and I’m also hungry, what am I going to eat the pills with… there being no food…It’s like that, you cannot take your pills without having food in your stomach, and you won’t have energy. (59 year-old woman, Khayelitsha)*

Older persons complained of tiredness, pain and hunger either because of having to walk to the facility or because of the time spent waiting at the facility. While most respondents felt that the quality of care was acceptable in general, many respondents said that when accessing their medication for chronic conditions, they experienced long queues and waiting times. At times, these issues led to missed doctor appointments and as a consequence, the respondent did not receive care.

A number of these barriers were partially eased by the format of HIV care and treatment distribution. Some respondents accessed HIV care through a “club”. The benefits of this program included contending with fewer people, shorter queues and a slightly better quality of service leading to interactions with the health system that respondents regarded as simpler and more efficient than for their other chronic medication. This interaction between the interviewer and an older female respondent alludes to the reduced barriers:

*R: Yes I just go there and I get that parcel, I don’t have to be in a queue, I just go straight to the Club…and hand in my card, then I’m given my pills and I walk out of the door.*

*I: So you are not delayed?*

*R: I don’t get delayed. (59 year-old woman, Langa)*

The findings above highlight additional problems with access to care that are posed for older persons living with HIV and one or more additional co-morbidities in accessing care within this context. These barriers are appeared to be connected particularly to care for their conditions other than HIV. One of the HIV service providers from Langa alluded to this problem in particular,

*Sometimes I don’t have to send [refer a person for something not treated here] because now what we are trying to do, we are trying to integrate everything like this ONE STOP SHOP… We are avoiding that because I think that’s one of the things that makes them default, this trip trap, but if we can just provide everything here then we will be able to monitor them closely. (HIV clinic nurse, Langa)*

As explained by the provider above, there is a move towards integration within the single clinic facility, in line with the Department of Health’s guidelines about integrated chronic disease management (ICDM) model [32]. The nurse at the HIV clinic at Vanguard described the use of a Chronic Care Club for those regardless of age who are stable and on ART and chronic medication; the group member accesses ART and NCD medications through the club. This Chronic Care Club is a synchromised group appointment approach very similar to the ART adherence Club where all required medications are dispensed to the patient through the Central Dispensing Unit (CDU) and individuals are monitored by a nurse and doctor [39]. Despite, this being an option at Vanguard, it was not mentioned by a single OPLWH in our sample, as they lived in Langa and access their HIV medications at the Langa HIV clinic. Despite being theoretically for any age group, it was unclear if the Chronic Care Clubs was meant for, tailored to, or accessed by older persons within the community.

## Discussion

The syndemic of HIV and NCDs within South Africa [5, 7, 12, 30, 40] is particularly problematic and pronounced for older persons who are likely to experience general ill health and particularly co-morbidities in the form of NCDs [12, 30, 41], along with social and economic vulnerability [19]. The patterns that emerged from this small-scale qualitative study, although not representative, seem to mirror those observed within epidemiological studies with the older persons reporting HIV and at least one additional chronic condition [3, 12]. This resulted in older persons’ having to seek care and access treatment for both their HIV and other conditions at different health facilities or with multiple health providers. Using the syndemics framing, it is, therefore, possible to see that the problems and barriers that older persons living with HIV and NCDs face within a non-integrated healthcare system can exacerbate their ill-health and perpetuate structural vulnerabilities [10].

All of those with HIV and an additional morbidity were engaged with care for their conditions. This linkage to care is encouraging and may be important for both future prevention and also older persons’ health and well-being [42, 43]. It could result from an ‘ART advantage’ [28]; these results and other research suggest that linkage to HIV care is associated with diagnosis and treatment of NCDs and other conditions [23, 26, 28, 29, 44].

The siloed provision of HIV and NCD care within the South African health system means that services are not integrated and their other conditions are not treated together [30, 32]. Further, it can also mean that providers are unaware of patients’ other conditions. One exception to bifurcated care is that of TB and HIV, another recognized syndemic with related biological risk and social and economic interactions [45, 46]. Due to this bifurcated nature of HIV and NCD care, older people with HIV experienced logistical problems that could present barriers to or result in delays in accessing care. Accessing care for two or more conditions, in two different sections of the health service meant our respondents had to deal with two distinctly organized appointments, even for those situated in one facility.

While health workers suggested that the option of streamlining appointments and various ‘clubs’ for accessing medication exist, our respondents did not seem to be aware of these options. Instead, they continued to access care through conventional means and dealt with seeking care for multiple conditions in different places, or on different days or times. This sometimes resulted in older persons feeling confused about where they were supposed to be for their treatment for each condition and at what time. These findings highlight the need, noted in other research, for improved communication between providers and patients and for the health service to be more responsive to and centred on the older patients’ needs [47–49].

Access to treatment and quality of care was relatively efficient within the HIV service, and ART adherence clubs allowed people to have group appointments. These aspects reduced burdens on both the health service and the patient, but only for those individuals seeking care for HIV and/or TB. This was not the case in the chronic care clinic and services, where in general, OPLWH were faced with many challenges such as long waits. Waiting times at clinics is another well-documented barrier to care, particularly for older persons [50].

In addition, these logistical barriers to access were compounded because of the challenge of having multiple appointments, and in some cases in a clinic situated in another community. Our findings support existing research that shows that physical barriers to care are associated with weakness, difficulty walking or symptoms for people with HIV in general [49] and specifically for older people [50–53]. Where distances or physical functionality affected older persons’ access to care on foot it was possible for them to use alternative means of transport but many experienced financial challenges to access also noted in other research [51, 52, 54]. In South Africa, social grants, particularly old age pensions, have been shown to facilitate access to healthcare for older persons [55] but in this study even where respondents were able *and* old enough to access a pension, it was insufficient to overcome all barriers to access. The financial barriers to care were intensified when multiple visits to healthcare in 1 month were required and therefore the costs were cumulative.

This study provides evidence for the complex interactions involved in the access to care for older persons who experience HIV and NCDs within this context. The results show the syndemic of HIV and NCDs can be exacerbated by the problems and barriers this population have with accessing care for the multiple conditions with which they are struggling [9, 10]. Mendenhall [9] argues that patient-centred care incorporating routine and diagnostic care can, at a single visit, save patients time and money, which in turn can improve outcomes and overall wellbeing. These were the exact complaints and desires outlined by our respondents, and thus are likely to address not just outcomes, but also the underlying social, economic, and psychological impediments to treatment.

The views of both patients and key informants have provided evidence for the potential benefits of integrated HIV and NCD care. The need for the integration of services has been widely acknowledged and a recent journal supplement very comprehensively explores both the need for and makes suggestions about possible ways in which integration within sub-Saharan Africa may be possible [56–58]. The need for integration is also recognised within South Africa [30, 34] and guidelines have been developed and the ICDM model tested [31, 32, 59] within South Africa, the roll-out of this system is slow. Despite evidence for some integrated practices such as the Chronic Care Club, the reality for our respondents was siloed services. Our interview respondents had limited understanding of the possibility for integration and limited ability to negotiate for it. Further, our results show that the physically split services in some districts present additional challenges to integration models and plans. Even in relatively well-run and well-resourced urban facilities, with well-trained staff, there are challenges.

Other geographical and provincial settings may present different or additional challenges not considered within this paper to the integration of chronic care for HIV and other conditions. Our sampling strategy, which mainly included sampling respondents through referrals from the HIV service and convenience sampling in the clinic, may have led us to miss individuals with fewer or greater barriers. Those with fewer barriers may have accessed HIV care through the Community Dispensing Unit or Community Health Workers. Those with greater barriers may not be attending services at all because they do not know their HIV status, are not adherent to their treatment or are too unwell to access services. In addition, our respondents may more likely to be people who prioritize their ART care, and the study may miss those who are being treated for NCDs who either do not know their HIV status or prioritize that care over HIV care. The sample is not representative and although attempts were made to ensure sampling of equal numbers of women and men and a similar proportion of respondents under and over 60 years of age, finding sufficient participants in each group was challenging. Older persons living with HIV may be even more likely to have co- or multi-morbidities with ill health consequently resulting in barriers in access to care for ***both*** HIV and NCDs, so in all likelihood, we have underestimated or understated the potential barriers and problems brought on by bifurcated care.

The problems associated with the minimal integration of health services for HIV and other conditions [30, 34, 60] are brought strongly into the spotlight by the findings of this study. This study confirms research that shows that older people living with HIV and other co-morbidities have many barriers to care [23, 25, 41, 61]. The complexity of ensuring access to care and delivering treatment for co-morbidities to those living with HIV is not a problem unique to older people, nor given the vertical nature of the HIV program in South Africa likely to be a specifically urban issue [50]. This study and other extant research, point to the need for implementation research that can help illuminate problems and test solutions that improve care for older persons and other populations. Despite the fact that older persons living with HIV may be more effectively linked into care and treatment as a result of their HIV status [23, 25], there is a need to assess and address bifurcated care. Integrating care through Chronic Care Clubs targeted at older persons, and making these group appointments available in conjunction with HIV care, could reduce the burden of multiple appointments, as well as waiting times at the clinic for older persons. In addition, improvements to and streamlining of these systems would reduce problems with access and empower older patients to negotiate care that meets their needs [47–49]. As noted by Lalkhen and Mash ([34]: 137) “patients also deserve their own goals, concerns and preferences to be taken into account.”

## Conclusion

In this urban South African community, older persons living with HIV and experiencing co-morbidities were faced with significant barriers to accessing healthcare because of the bifurcated nature of care. Integration of care needs to address the needs of older populations, not just those 15–49 years old, which most often focus on TB as a co-morbidity. As older persons age with HIV or are newly diagnosed in this context, the numbers of people within this vulnerable population increases, as does their need for ART alongside treatment for a complex array of other medical conditions. The syndemic nature of HIV and NCD co-morbidity for older persons, exacerbated by the structural and systemic problems they encounter, means that accessing bifurcated care poses a challenge to their retention in care. Health policy needs to address the integration of HIV and NCD care as the population living with HIV continues to age, increasing the likelihood that they will also be living with other chronic conditions including NCDs.
